# Knowledge, Attitudes, and Practices of Parents Regarding Antibiotic Use for Acute Upper Respiratory Tract Infections in Children in Basrah, Iraq

**DOI:** 10.7759/cureus.70996

**Published:** 2024-10-07

**Authors:** Abrar I Albadr, Israa I Albadr

**Affiliations:** 1 Department of Biochemistry, College of Medicine, University of Basrah, Basrah, IRQ; 2 Department of Family Medicine, Al-Aqsa Intifada Center, Basrah, IRQ

**Keywords:** antibiotics, bacterial resistance, children, parent knowledge, upper respiratory tract infection

## Abstract

Background: Upper respiratory tract infections (URTIs) are mainly caused by viral infections, but parental attitudes often lead to inappropriate antibiotic prescriptions, thereby increasing antibiotic resistance. This study aimed to evaluate parents' knowledge, attitudes, and practices (KAP) regarding antibiotic use for children with URTIs in Basrah, Iraq.

Methods: A cross-sectional study was conducted at 14 primary healthcare centers in Basrah from November 2023 to April 2024. A questionnaire was designed and administered to determine parents' KAP regarding antibiotic use for children suffering from URTIs.

Results: Four hundred parents completed the questionnaire. The majority (81%) of parents were aware that antibiotic misuse leads to bacterial resistance. Sixty percent of respondents disagreed that viral infection is a leading cause of URTI and that it can be self-limited without antibiotics. A total of 67.6% of the parents were aware of the possibility of having side effects from antibiotic consumption. Moreover, more than half of parents (58.8%) never asked their pediatricians for an antibiotic prescription for their children.

Conclusion: Educational interventions are necessary to raise parents' awareness of antibiotics to reduce inappropriate use and its consequences.

## Introduction

Antibiotic resistance has become an important public health problem due to the increasing spread of highly resistant bacteria and the decline in the discovery of new antibiotics in recent decades [1].

As antibiotic resistance increases, physicians are less likely to be able to treat infections because of the lack of therapeutic options available to them [2]. Currently, there is no comprehensive solution to this problem [3,4].

Prescription of antibiotics is common in pediatrics for upper respiratory tract infections (URTIs), which are mostly viral in nature and often self-limiting [5]. It has been shown that even when common RTIs are caused by bacteria, they are likely to resolve without antibiotics [6]. Unnecessary antibiotic prescription is the main driver for antibiotic resistance [1].

Parents and pediatricians share responsibility for this antibiotic misuse, which is contributing to antibiotic-resistant strains of bacteria [1]. Whether an antibiotic is prescribed is heavily influenced by parents' beliefs and expectations. When parents panic over their children's acute illnesses, they visit their pediatricians more frequently expecting or demanding antibiotic prescriptions, leading to unnecessary antibiotic use. In addition, physicians are under pressure to meet patients' expectations, which leads them to prescribe antibiotics for viral URTI. Therefore, parents' knowledge of antibiotics used to treat URTIs can therefore greatly reduce antibiotic resistance [7-10]. Various studies conducted in different countries have indicated that parental knowledge of antibiotic use is variable [1,11-13].

There is a lack of knowledge among parents regarding antibiotic use in relation to URTIs in studies from Jordan, Saudi Arabia, and the United Arab Emirates (UAE) [13-15].

Contrary to this finding, a study from Cyprus found that antibiotic overuse wasn't caused by parents' poor knowledge but by doctors' decisions [12]. There were similar findings in Greece, where doctors' over-prescription of antibiotics led to antibiotic misuse [1].

This study aims to assess parents’ knowledge, attitudes, and practices (KAP) regarding the use of antibiotics for URTIs, to raise awareness, and to prevent unnecessary antibiotic use by making the right interventional educational programs.

## Materials and methods

We conducted a cross-sectional survey in 14 primary health care (PHC) centers in Basrah governorate between November 2023 and April 2024. To obtain a representative sample of parents, PHC centers were chosen based on geographic clustering sampling. Participants in the current study were adults with children under 12 years old attending the selected healthcare centers during the study period; eligibility criteria were determined during registration at the reception.

We selected both fathers and mothers to participate; however, parents who didn't speak Arabic or English, those with learning disabilities/dementia, and those who couldn't complete the questionnaire were excluded from the study.

An online sample size calculator (Raosoft Inc., Seattle, USA) was used to determine the sample size based on an estimated response distribution of 50%, a margin of error of 5%, and a 95% confidence interval. Considering the data was collected face-to-face, 384 participants were calculated as the final sample size based on a 100% response rate. To prevent data loss or non-response rates, the sample size was increased to 400.

Participants were interviewed face-to-face in the waiting area using a pre-tested questionnaire. The questionnaire had been validated previously for use in a similar study in Greece [1]. We used an Arabic version of the questionnaire, adapted to ensure that the questions were applicable to the local population.

A total of four sections (A, B, C, and D) comprised the final questionnaire. Section A focused on sociodemographic information about participants, including their age, gender, educational level, monthly income, number of children, and their ages, as well as whether the child suffered from chronic diseases such as asthma. Section B included questions regarding parental knowledge of antibiotics. They were asked to mark antibiotic names out of five commonly used medications and to answer questions regarding antibiotics' general use, side effects, and their use in viral infections. Furthermore, section B explored sources of information regarding the use of antibiotics. Section C studied parents' expectations and attitudes about pediatricians prescribing antibiotics to their children with URTIs, including their attitude toward antibiotic use without pediatrician advice and factors that affect it. Finally, section D illustrated parents’ answers to questions linked to the medical practice. Parents were asked to indicate if their pediatrician spends adequate time elucidating the illness and suggesting antibiotic treatment for a child’s illness, and if he/she is affected by their demand to prescribe antibiotics for their child. Most questions were based on the 5-point Likert scale, which expresses emotions: strongly disagree, disagree, uncertain, agree, and strongly agree; frequency: always, most of the time, often, sometimes, and never.

KAP scores were calculated by summing up the Likert-scale responses for each category.

Statistical analysis

The data were analyzed using IBM SPSS Statistics for Windows, Version 26 (Released 2019; IBM Corp., Armonk, New York, USA). The analysis of answers to questions involved descriptive statistics, e.g., frequency and percentage for categorical variables and means ± standard deviation (SD) for numerical variables.

## Results

The study included 400 parents (response rate: 100%). Most of the participants were females (n = 332; 83%), and the mean age was 32.6 ± 8.9. The majority of parents had achieved elementary education as their highest level of schooling (155; 38.8%). Approximately half of the participants (n = 217; 54.3%) had children under six years of age. Only 57 (14.2%) had a child with chronic illness (i.e., asthma). The sociodemographic characteristics of participants are shown in Table 1.

**Table 1:** Parents' demographic characteristics (n = 400) ª IQD: Iraqi Dinar

Demographic characteristics	Frequency or mean ± SD	Percentage
Gender		
Female	332	83%
Male	68	17%
Age ± SD (year)	32.6 ± 8.9	
Age of children in years		
<6	217	54.2%
6-12	183	45.8%
Participant's educational level		
Illiterate	45	11.2%
Elementary school	155	38.8%
High school	96	24.0%
University	104	26.0%
Income level of the family per monthª		
Low (less than 500 000 IQD)	140	35%
High (more than 500 000 IQD)	260	65%
Residency		
Urban	400	100%
Rural	-	
Child with chronic illness	57	14.2%

Knowledge

The majority of parents (47.6%) reported that their physician was their primary source of information about antibiotics. The pharmacist was the second most common source at 18.3%, followed by television and social media at 17%. Other less common sources included books (3.3%) and family members/friends (0.5%). However, 13.3% of parents stated that they had never received any information about antibiotics from any of these sources. The parents were asked to identify antibiotics from antipyretics, bronchodilators, and expectorants in a list of drugs; most parents (56.4%) were able to identify that amoxicillin was an antibiotic. Moreover, 21.5% and 12.3% of parents identified cough syrups and paracetamol, respectively, as antibiotics. Only 6.3% and 3.5% identified allermin and ibuprofen as antibiotics. Table 2 demonstrates the responses to questions related to knowledge. About 81% of parents are aware that antibiotic misuse causes bacteria to develop resistance; however, 50.1% are still willing to give their children antibiotics because they believe it will cause their children to recover faster. In fact, 60% of respondents disagreed that viral infection is a leading cause of URTI and that it can be self-limited without antibiotics. Moreover, 22.1% of parents believe that antibiotics should be administered to any child with a fever. A total of 67.6% of the parents were aware of the possibility of having side effects from antibiotic consumption, and 69.6% of parents believe that antibiotics can help prevent complications from URTIs. Finally, 70.8% of parents thought that stronger antibiotics were constantly being developed. Reported side effects of antibiotic use were stomach upset (n = 97; 24.2%), decreased immunity (n = 81; 20.3%), nephrotoxicity (n = 66; 16.5%), hepatotoxicity (n = 50; 12.5%), hypersensitivity reactions (n = 45; 11.2%), and increased resistance (n = 5; 1.3%); also (n = 56; 14%) of parents were unsure or did not report any side effects.

**Table 2:** Parental knowledge regarding antibiotic use in children with URTIs (n = 400) Questions adopted from Panagakou et al. [1] URTIs: upper respiratory tract infections

Question statement	Item	Frequency	Percentage %
All children with fever should be treated with antibiotics	Strongly agree	35	8.8
	Agree	53	13.3
	Disagree	249	62.1
	Strongly disagree	39	9.8
	Uncertain	24	6.0
Antibiotics speed up the recovery of children with flu-like symptoms	Strongly agree	33	8.3
	Agree	167	41.8
	Disagree	140	35.0
	Strongly disagree	23	5.8
	Uncertain	37	9.1
Due to the fact that many upper respiratory tract infections (such as colds, flu, sore throats, ear infections) have a viral origin, antibiotics should not be used as they are self-limiting.	Strongly agree	44	11
	Agree	99	24.8
	Disagree	218	54.5
	Strongly disagree	22	5.5
	Uncertain	17	4.2
There are no side effects associated with antibiotics	Strongly agree	25	6.3
	Agree	70	17.5
	Disagree	247	61.8
	Strongly disagree	23	5.8
	Uncertain	35	8.6
When antibiotics are misused, their efficacy is reduced and bacterial resistance is increased	Strongly agree	73	18.3
	Agree	251	62.7
	Disagree	41	10.3
	Strongly disagree	2	0.5
	Uncertain	33	8.2
Antibiotic use can prevent complications from upper respiratory tract infections	Strongly agree	79	19.8
	Agree	199	49.8
	Disagree	67	16.8
	Strongly disagree	22	5.5
	Uncertain	33	8.1
New antibiotics can be developed to kill bacteria that have become resistant to existing antibiotics	Strongly agree	125	31.3
	Agree	158	39.5
	Disagree	14	3.5
	Strongly disagree	20	5.0
	Uncertain	83	20.7

Attitude

**Figure 1:**
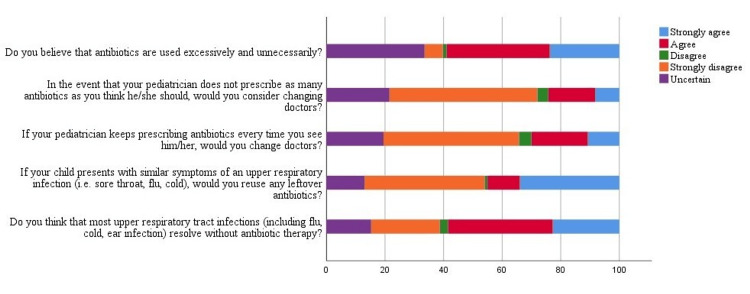
Percentage of parents' responses to questions related to attitude Questions adopted from Panagakou et al. [1]

Figure 1 shows how parents view the use of antibiotics for URTIs. Over half of parents (n = 236; 59.1%) agreed that antibiotics are frequently prescribed without proper justification, and 54.3% (n = 217) were unlikely to change pediatricians if they were not easily prescribed antibiotics. However, 30% (n = 120) said they would change pediatricians because they prescribe antibiotics easily. Also, 45% (n = 180) of parents agreed that they would use the same antibiotics as before for their children's same symptoms. Last, 26.3% (n = 105) of respondents believed URTIs are not self-limited.

Practice

**Figure 2:**
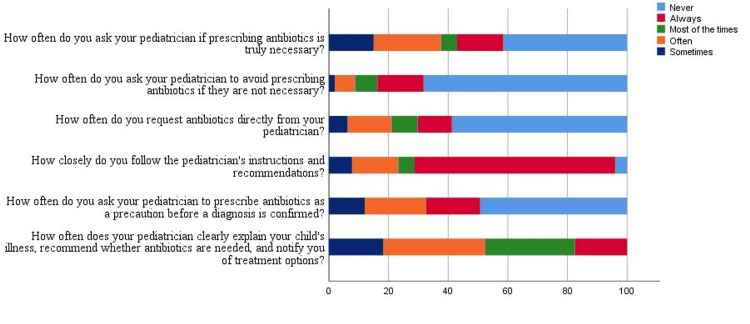
Percentage of parents' responses to questions related to practice Questions adopted from Panagakou et al. [1]

There were approximately 47.5% (n = 190) of parents who were satisfied with their pediatricians because they provided sufficient information about their children's diagnosis and treatment plan. More than half of parents (n = 235; 58.8%) never asked their pediatricians for an antibiotic prescription for their children. Moreover, the majority of parents (n = 269; 67.3%) follow their pediatrician's instructions strictly. Approximately 21% (n = 84) of parents would ask their pediatrician whether antibiotics should be administered. About 23% (n = 92) of parents would ask their pediatrician not to prescribe antibiotics if they did not need them. However, a precautionary prescription for antibiotics would be requested by 38.8% (n = 155) of parents before a diagnosis was confirmed by their pediatrician. Parents' practices regarding antibiotic use in URTI are shown in Figure 2.

## Discussion

We conducted this study to determine knowledge and attitudes about antibiotic use and practices in the management of childhood URTIs in a sample of parents from Basrah, Iraq.

This study demonstrated that parents and pediatricians have a trusting relationship. In cases of URTIs in children, most parents trust the information and prescriptions provided by their pediatricians, and only a few parents would switch pediatricians if they were over- or under-prescribed antibiotics. Furthermore, 67.3% of parents stated that they adhere exactly to their pediatrician's recommendations, and almost half stated that their pediatrician was the main source of information about antibiotic use and misuse. Similar KAP studies identified pediatricians as the primary source of information about antibiotic use or misuse [1,16], whereas Xiang et al. identified media (e.g., television) as the main source of such information [17]. In the present study, as in other studies [15,16,18] many participants did not understand the appropriate use of antibiotics or had misconceptions about doing so. A total of 22.1% of parents thought that every child with a fever needed antibiotics, consistent with a Saudi Arabian study of 19%, possibly because of their apprehension of the dire consequences of fever and inadequate knowledge [5]. Furthermore, 69.6% of parents believe antibiotics will reduce complications from URTIs. This belief is evident in their attitudes since their antibiotic expectations were high for certain URTIs. Similar to previous research, the majority of parents were aware that antibiotic misuse results in bacterial resistance; at the same time, 70.8% of them also believed that scientists would always be able to develop effective antibiotics for resistant bacteria even if resistance arose from inappropriate use, which might explain their overuse of antibiotics [13]. Sixty percent of parents in the current study did not agree that URTIs are mainly viral in origin and are self-limiting without the need for antibiotics, which is in contrast to Greek parents, of which 80% believed that URTIs are mostly self-limited [1]. Our findings showed that 45% of parents in the current study reuse leftovers and share antibiotics with their children, while a cross-sectional KAP study in Malaysia showed that 15% of Malaysian parents reused leftovers and 24% shared antibiotics [19]. In the current study, 59.1% of parents thought antibiotics were used unnecessarily and too much. Similar findings were reported by Panagakou et al. (78%) and Rousounidis et al. (81%) [1,12].

This study had strengths as well as limitations. While the response rate was 100%, likely due to face-to-face interviews that may have helped to establish a stronger rapport with respondents. However, the study was only conducted at health centers in Basrah; therefore, the findings might not be representative of other regions in Iraq. Additionally, the data were collected from parents who attended PHC centers, which limits their generalizability to other types of healthcare services, such as the private sector. Furthermore, parents were also asked several questions about their previous experiences and antibiotic use, which may have contributed to recall bias.

## Conclusions

Generally, parents in Basrah have a high level of confidence in their doctors when it comes to prescribing antibiotics to their children. However, they also demonstrated a lack of adequate knowledge about the use of antibiotics in cases of URTIs. It is urgent to develop educational initiatives and health campaigns aimed at promoting rational antibiotic usage.
